# Gut Microbiota and Fecal Metabolites Associated With Neurocognitive Impairment in HIV-Infected Population

**DOI:** 10.3389/fcimb.2021.723840

**Published:** 2021-10-25

**Authors:** Ruihua Dong, Haijiang Lin, Xiaoxiao Chen, Ruizi Shi, Shiying Yuan, Jing Li, Bowen Zhu, Xiaohui Xu, Weiwei Shen, Keran Wang, Xiao-Ou Shu, Ding Ding, Na He

**Affiliations:** ^1^ Department of Nutrition and Food Hygiene, School of Public Health, Fudan University, Shanghai, China; ^2^ Department of Epidemiology, School of Public Health and The Key Laboratory of Public Health Safety of Ministry of Education, Fudan University, Shanghai, China; ^3^ Department of Epidemiology, Taizhou City Center for Disease Control and Prevention, Taizhou, China; ^4^ Division of Epidemiology, Department of Medicine, Vanderbilt Epidemiology Center, Vanderbilt University Medical Center, Nashville, TN, United States; ^5^ Institute of Neurology, Huashan Hospital, Fudan University, Shanghai, China; ^6^ Shanghai Institute of Infectious Diseases and Biosecurity, Fudan University, Shanghai, China

**Keywords:** HIV, neurocognitive impairment (NCI), gut microbiota, butyrate-producing bacteria, metabolomics, vitamin D

## Abstract

Gut microbiota dysbiosis has been associated with many neurological diseases. However, how microbiota composition and metabolism relate to neurocognitive impairment (NCI) in HIV-infected individuals is largely unknown. In this study, a total of 102 HIV infected participants were classified into two groups—those with NCI and those without—using the global deficit score (GDS). Fecal samples were collected from the participants for 16S rRNA gene sequencing and untargeted metabolomics. The plasma level of 25 hydroxy-vitamin D (25(OH)D) was also evaluated. Although α-diversity and β-diversity were comparable, the HIV patients with NCI were significantly different from those without NCI in terms of abundance of several gut microbiota. The decreased abundance of butyrate-producing bacteria (BPB) and increased abundance of *Klebsiella* were related with NCI and carotid intima-media thickness (CIMT). Significant differences in fecal metabolites were also found between individuals with *versus* without NCI, including increased bile acids and bioactive lipids, decreased vitamin D, terpenoids, and resolvin D1 in the NCI group. Furthermore, the perturbed metabolic profile was closely related to BPB and *Klebsiella*. In addition, a low level of vitamin D was associated with NCI and CIMT. Both fecal and plasma vitamin D were positively correlated with BPB. Our results show that BPB and *Klebsiella* and the associated metabolites are associated with NCI in people with HIV. In addition, vitamin D, both in feces and blood, was associated with NCI and BPB, suggesting a protective effect of vitamin D on NCI.

## Introduction

HIV is neurovirulent and frequently causes brain impairment (Williams et al., 2020). Therefore, HIV-infected individuals often experience a neurocognitive impairment (NCI), referred to as HIV-associated neurocognitive disorders (HAND) (Heaton et al., 2010). The CNS HIV Antiretroviral Therapy Effects Research study has reported a prevalence of 35 and 50% of older adults living with HIV who experience mild to moderate cognitive impairment (Ding et al., 2017a; Ding et al., 2017b). The possible reasons for the persistent prevalence of NCI include accelerated aging of HIV population, direct damage from the virus, and neurotoxicity of specific antiretroviral drugs (Qiao et al., 2019). However, the pathogenesis of NCI in HIV-infected individuals still remains incompletely understood.

Gut microbiota dysbiosis is common in HIV-infected individuals, including decreased diversity and alterations in gut microbiome composition compared with healthy individuals (Ashuro et al., 2020). In HIV-infected individuals, loss of intestinal barrier integrity, an increased plasma level of microbiota products, and systemic inflammation have been correlated with NCI (Zhang et al., 2019). Furthermore, one investigation found that the HAND group presented a significantly lower α-diversity compared with the non-HAND group, using the 16S rRNA gene sequencing approach, suggesting a potential relationship between the gut microbiota and HAND (Zhang et al., 2019). Therefore, gut microbiota dysbiosis may play a role in the pathogenesis of NCI.

Of note is that non-targeted fecal metabolomics studies have been used in understanding metabolites associated with gut microbiota alteration in complex disease development (Luan et al., 2019)—for example, a study found distinct changes in the fecal metabolites of depressive rats, and the gut microbiota was altered in association with fecal metabolites (Yu et al., 2017). Nevertheless, no study has been conducted using integrative analysis of fecal metabolome and microbiome to understand the pathogenesis of NCI.

To explore the role of gut microbiota and the underlying metabolic mechanisms in the development of NCI among HIV-infected individuals, we applied 16S rRNA gene sequencing and non-targeted fecal metabolomics to compare gut microbiota and fecal metabolite differences among HIV-infected individuals with and without NCI.

## Materials and Methods

### Study Settings and Design

The study participants were recruited from the Comparative HIV and Aging Research in Taizhou (CHART). The detail of the prospective cohort has been described previously (Ding et al., 2020). Between February and December 2017, 1,770 HIV-positive adults and 3,350 HIV-negative controls were enrolled from Taizhou Prefecture of Zhejiang Province, Eastern China. The samples of this study came from one of the substudies under the CHART cohort. The substudy included HIV-infected patients if they were between 40 and 80 years, able to communicate in Mandarin and literate, and had no serious hearing or eye problem. The included participants further took a comprehensive battery of neuropsychological (NP) test (supplementary materials) by certified professionals. The global cognitive score was defined by the global deficit scores (GDS) dichotomized as impaired (GDS ≥ 0.5) or unimpaired (GDS < 0.5) (Cysique et al., 2007). In total, 385 HIV-infected patients and 411 controls completed the NP tests. Among them, 122 HIV-infected patients were identified with NCI, and 122 controls (HIV-infected patients without NCI) were randomly selected, with comparable age and gender. In 2019, we invited these participants to take the NP test again and provide their fecal samples. Then, 88 HIV-infected patients with NCI and 47 controls agreed to participate in this study and completed the NP test. After excluding the participants without fecal samples, a total of 67 HIV-infected persons with NCI and 35 HIV-infected persons without NCI were included in the present analysis (**Supplementary Tables S2** and **S3**) The Ethics Committee of the School of Public Health at Fudan University approved this study. All individuals gave informed consent at enrollment.

### Data Collection

For the baseline survey, all cohort participants were administered with a questionnaire interview to collect basic demographics including age, sex, education, lifestyle, *etc*. Physical examinations of height, weight, and blood pressures (BP) were carried out. BP was measured twice, and the average of the two readings were recorded. Body mass index (BMI) was calculated as body weight divided by the height squared (kg/m^2^) of the participant. Hypertension was defined as systolic BP ≥140 mmHg or diastolic BP ≥90 mmHg. They also received comprehensive physical and biochemical examinations and B-mode ultrasonic and electrocardiographic examinations (Qiao et al., 2019). The most recent CD4 T cell count was extracted from the National HIV/AIDS Comprehensive.

Neurocognitive performances were assessed using the NP test (**Supplementary Materials**) by certified professionals. The global cognitive score was defined by the GDS dichotomized as impaired (GDS ≥0.5) or unimpaired (GDS <0.5) (Cysique et al., 2007). Depressive symptoms were measured by the 10-item version of Zung Self-Rating Depression Scale (Zung, 1973). The insomnia symptoms were measured based on Jenkins’s four-item sleep questionnaire (Jenkins et al., 1988). Frailty phenotype was assessed using the Fried criteria, with the exception of physical activity, for which we used the proxy described by Onen et al. (Ding et al., 2017a; Ding et al., 2017b).

### 16S rRNA Sequencing and Measurement of Fecal Metabolomics

We collected fecal samples from 102 participants of this study. The fecal samples were collected in a feces container and stored at −80°C. Total genomic DNA was isolated using DNA Extraction Kit (Qiagen, Düsseldorf, Germany) as per the instructions of the manufacturer. Details of the 16S rRNA sequencing and the quantitative measurement of fecal metabolomics as well as relevant data analysis are available in the **Supplementary Materials**.

### 25 Hydroxy-Vitamin D [25(OH)D] Measurements

The plasma samples were refrigerated immediately after phlebotomy and centrifuged and frozen in a central laboratory within 2 h. Levels of 25(OH)D were measured using enzyme-linked immunosorbent assays and categorized as normal (30 ng/ml), insufficient (20 to 29 ng/ml), and deficient (<20 ng/ml) (Manion et al., 2017).

### Statistical Analyses

The demographic factors and clinical parameters were compared between the two groups. Continuous variables were presented as median (interquartile range) or mean (standard deviation) based on whether the data were normally distributed. Categorical variables were summarized as numbers and percentages (%).

The α-diversity indices, including Chao, Shannon, and Simpson, were calculated (Sun et al., 2016). Weighted and unweighted UniFrac distances were used to measure β-diversity (Ji et al., 2018). Differences in characteristics and diversity indices between groups were compared by Student’s *t*-test (continuous variables, normal distribution), the Mann–Whitney *U*-test (continuous variables, skewed distribution), or chi-square test (categorical variables). Differences in the relative abundances of gut microbiota at the genus level between groups were assessed using a generalized linear model with sex, age, CD4 count, sexual preference, and BMI, included as co-variates. We further identified the differentially abundant taxa in the NCI and non-NCI groups by linear discriminant analysis effect size (LEfSe) (Li et al., 2019).

We performed orthogonal projection to latent structure-discriminant analysis (OPLS-DA) to examine the overall microbial metabolite distribution between the two groups. The fit and predictability of the models obtained were determined by the *R* ^2^ *Y* and *Q* ^2^ values, respectively. In the OPLS-DA model, we generated the variable importance plot (VIP) to select potential biomarkers. Depending on the distribution of the data, correlations between clinical parameters, fecal metabolites, 25(OH)D, and genera abundance were calculated by Spearman’s rank test. Statistical significance was defined as two-sided and *P <*0.05. The *P*-values were adjusted to control the false discovery rate (FDR). OPLS-DA was performed with the software SIMCA-P+, version 14.0 (Wan et al., 2019). All other statistical analyses were performed in R, version 3.6.2, and R packages (pheatmap, ggplot2, ggrepel, ropls, corrplot, and status) were applied in this work.

## Results

### Characteristics of the Study Participants

**Table 1** presents the demographics, clinical symptoms, and laboratory biomarkers of the 102 participants. There are no significant differences in the demographic measurements between the two groups. The HIV-infected participants in the NCI group had higher left and right carotid intima-media thickness (CIMT) than those in the non-NCI group. The NCI group also had a higher proportion of carotid plaques and cholesterol crystal than those in the non-NCI group (**Table 1**).

**Table 1:** Characteristics of the NCI and non-NCI groups (*n* = 102).

Characteristics	NCI group (*n* = 67)	Non-NCI group (*n* = 35)	*P-*values
Demographics			
Sex, *n* (%)			
Male	55 (82.1)	25 (71.4)	0.186
Female	12 (17.9)	10 (29.4)	
Age, years, (mean ± SD)	54.6 ± 9.7	55.3 ± 9.3	0.708
BMI, kg/m^2^ (mean ± SD)	23.2 ± 3.0	22.9 ± 2.6	0.679
Education, *n* (%)			
≤Primary school	49 (70.0)	20 (49.0)	0.297
Middle school	13 (19.4)	11 (31.4)	
≥High school	5 (7.5)	4 (8.6)	
Current smoker, *n* (%)			
Yes	35 (52.2%)	13 (37.1%)	0.107
No	32 (47.8%)	22 (62.9%)	
Current alcohol use, *n* (%)			
Yes	26 (38.8%)	14 (40.0%)	0.956
No	41 (61.2%)	21 (60.0%)	
HIV-related characteristics			
Time since HIV diagnosis, years, (median, IQR)	5.2 (3.8, 8.2)	4.8 (3.2, 7.5)	0.782
Duration on cART, years (median, IQR)	4.3 (3.7, 6.3)	4.0 (2.9, 7.6)	0.578
Sexual preference (homosexual, %)	14 (20.9%)	9 (25.7%)	0.554
Current CD4 count, cells/μl (mean ± SD)	414.9 ± 194.9	472.2 ± 230.5	0.215
Clinical symptom			
Hypertension	32 (47.7%)	15 (42.8%)	0.572
History of diabetes	6 (9.0%)	2 (5.7%)	0.569
Depressive symptoms	27 (40.3%)	13 (37.1%)	0.759
Insomnia symptoms	13 (19.4%)	5 (14.3%)	0.510
Frailty symptoms	18 (26.9%)	10 (28.6%)	0.858
Laboratory biomarkers			
HDL (median, IQR)	1.2 (0.9, 1.5)	1.2 (1.0, 1.5)	0.736
LDL (median, IQR) CHOL (median, IQR)	2.3 (1.8, 2.9) 4.7 (3.9, 5.4)	2.5 (2.1, 3.0) 4.5 (3.9, 4.9)	0.401 0.242
TG (median, IQR)	2.0 (1.2, 3.2)	1.7 (1.0, 3.0)	0.371
Left CIMT (median, IQR)	1.1 (0.7, 1.4)	0.9 (0.8, 1.0)	0.017^*^
Right CIMT (median, IQR)	1.2 (0.8, 1.2)	0·9 (0.8, 1.0)	0.008^**^
Carotid plaques, *n* (%)			
Yes	36 (53.7%)	9 (25.7%)	0.007^**^
No	31 (46.3%)	26 (74.3%)	
Cholesterol crystal, *n* (%)			
Yes	20 (29.9%)	1 (2.9%)	0.001^**^
No	47 (70.1%)	34 (52.2%)	

NCI, neurocognitive impairment; BMI, body mass index; CIMT, carotid intima-media thickness; IQR, interquartile range; SD, standard deviation.

*P < 0.05; **P < 0.01.

### Gut Microbiota Diversity Index and Gut Microbiota Composition

**Figure 1:**
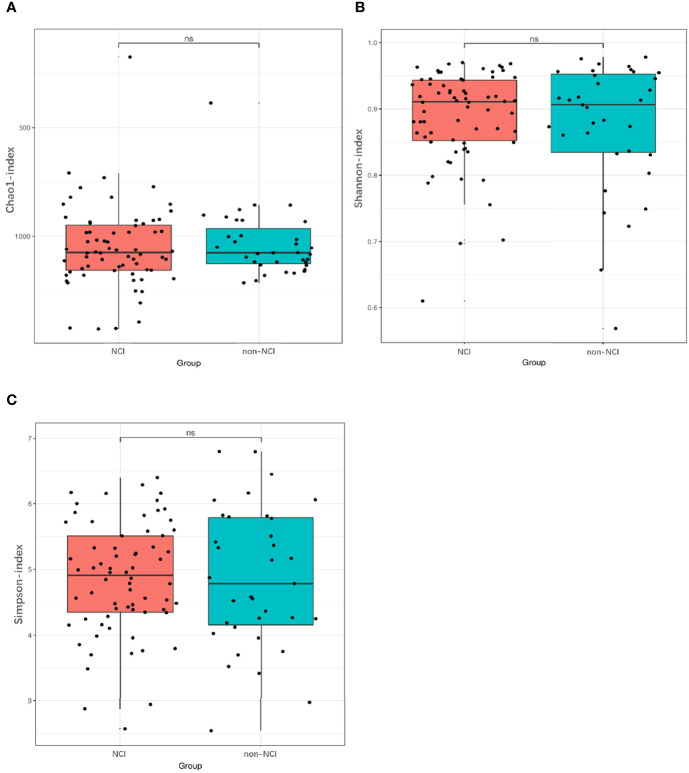
Differences in α-diversity of gut microbiota between NCI and non-NCI groups. NCI group, HIV-infected persons with neurocognitive impairment; non-NCI, HIV-infected persons without neurocognitive impairment; ns, not significantly different α-diversity of gut microbiota from the NCI group compared with the non-NCI group. Statistical significance was set at *P* = 0.05. **(A)** Chao1 index, **(B)** Shannon index, and **(C)** Simpson index.

**Figure 2:**
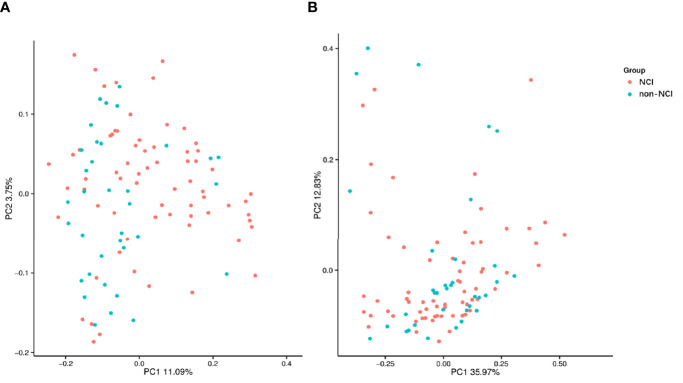
Unweighted **(A)** and weighted **(B)** analyses of similarities and principal coordinate analysis based on the distance matrix of UniFrac dissimilarity of the fecal microbial communities in the NCI and non-NCI groups. NCI group, HIV-infected persons with neurocognitive impairment; non-NCI, HIV-infected persons without neurocognitive impairment; PC, principal components. Each symbol represents a sample.

An analysis of the α-diversity showed that the Shannon, Simpson, and Chao indices were not significantly different between the NCI and non-NCI groups (Shannon index, *P* = 0.95; Simpson index, *P* = 0.90; and Chao index, *P* = 0.73; **Figures 1A–C**). The β-diversity of gut microbiota was evaluated based on the unweighted UniFrac distance matrix (*P* = 0.082), and the weighted UniFrac distance matrix (*P* = 0.459) showed no differences between the two groups in the fecal microbial communities (**Figures 2A, B**).

**Figure 3:**
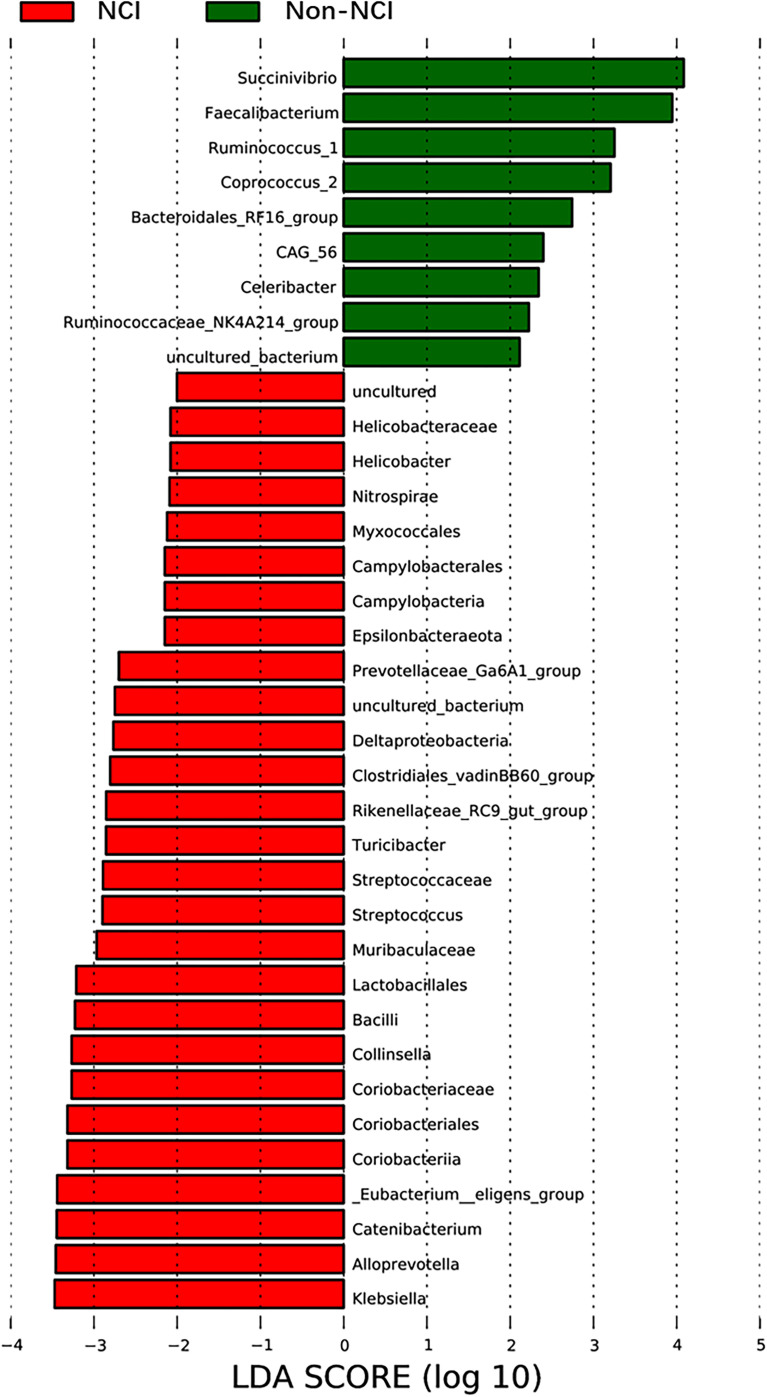
LEfSe was used to identify the most differentially abundant taxa of bacterial communities between the NCI and non-NCI groups, with LDA score >2 (b, d). NCI group, HIV-infected persons with neurocognitive impairment; non-NCI, HIV-infected persons without neurocognitive impairment; LEfSe, linear discriminant analysis effect size; LDA, linear discriminant analysis.

The species abundance of Spirochaetes and Epsilonbacteraeota was higher in the NCI group than in the non-NCI group at the phylum level. In addition, 56 genera showed significant differences between the two groups. The top 20 most abundant genera are presented in **Supplementary Table S4**. Only the differences in the abundance of Coprococcus_2, Treponema_2, Pseudomonas, and Thermopolyspora were significant between the two groups after FDR correction. LEfSe identified 36 discriminative features whose relative abundance varied significantly between the two groups. At the genera level, the microbiota of the non-NCI group was enriched with nine genera, while the NCI group was enriched with 27 genera (**Figure 3**). We conducted the following correlation analyses in the full cohort using the top 20 genera with the most abundant difference.

### Correlation Between the Abundance of Genera With Clinical Parameters

*Streptococcus* (*P* = 0.014) and Treponema_2 (*P* = 0.020) were inversely correlated with CD4 count. *Faecalibacterium*, Ruminococcus_1, Coprococcus_2, and Ruminococcaceae_NK4A214_group were inversely correlated with the left CIMT, while *Klebsiella* was positively correlated with the left CIMT. *Faecalibacterium* was inversely correlated with right CIMT (*P* = 0.018), while *Klebsiella* was positively correlated with the right CIMT (*P* = 0.018). However, we did not find a difference in FDR-corrected comparison (**Supplementary Table S5**). No significant relationship was found between the abundance of genera and other clinical parameters.

### Distinct Differences Between Fecal Metabolites in the NCI Group and Non-NCI Group

The OPLS-DA model showed strong differences in the overall microbial metabolite distribution between the NCI and the non-NCI groups (*R* ^2^ *Y* = 0.782, *Q* ^2^ = 0.215, **Supplementary Figure S3**). Twenty-four metabolites were identified as potential biomarkers. **Supplementary Table S6** presents the corresponding retention time, m/z, and VIP values.

Fourteen metabolites [bile acids (BAs), glycerophosphoinositols, fatty acids, eicosanoids, and fatty amides] were significantly increased in the NCI group compared to the non-NCI group. Four vitamin D metabolites [1alpha,25-dihydroxy-2alpha-(3-hydroxypropoxy)-19-norvitamin D3, (20S)-24-hydroxy-19-norgeminivitamin D3, 24,24-difluoro-1,25,26-trihydroxyvitamin D3, and atocalcitol], five terpenoids [astaxanthin diglucoside/astaxanthin β-D-diglucoside, lutein, tangeraxanthin, (-)-fusicoplagin A, and 18alpha-hydroxyglycyrrhetic acid], and resolvin D1 were significantly decreased in the NCI group compared with the non-NCI group (**Supplementary Table S6**).

### Correlation of the Gut Microbiota and Fecal Metabolites

**Figure 4:**
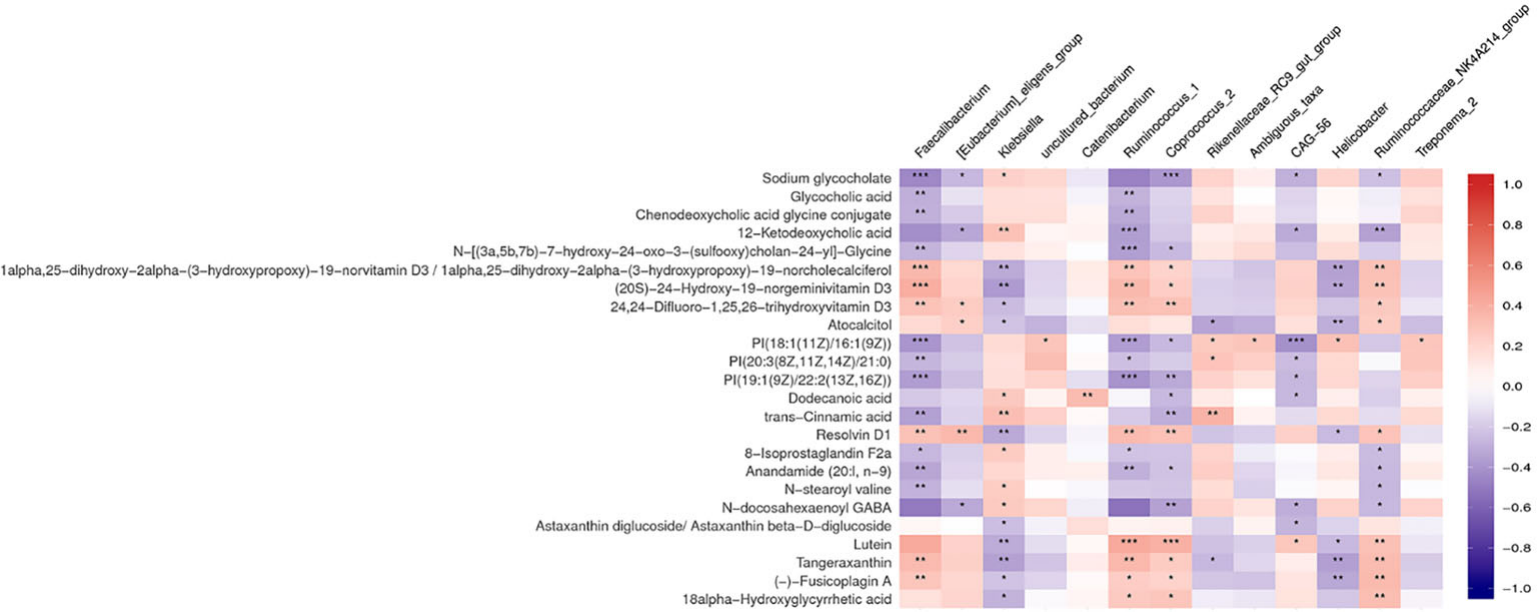
Heat map summarizing the correlation of perturbed gut microbiota genera and altered fecal metabolites between the NCI and non-NCI groups. *P < 0.05; **P < 0.01; ***P < 0.001.

Multiple correlations were found between fecal metabolites and specific gut bacteria (**Figure 4**). The metabolites which increased in the NCI group were consistently negatively correlated with *Faecalibacterium*, Corprococcus_2, and Ruminococcus_1 and positively correlated with *Klebsiella*. The metabolites which decreased in the NCI group, including four vitamin D metabolites, five terpenoids, and resolvin D1, were positively correlated with *Faecalibacterium*, Corprococcus_2, and Ruminococcus_1, whereas they were negatively correlated with *Klebsiella*.

### Correlation Between Plasma 25(OH)D With Clinical Parameters and Gut Microbiota

**Figure 5:**
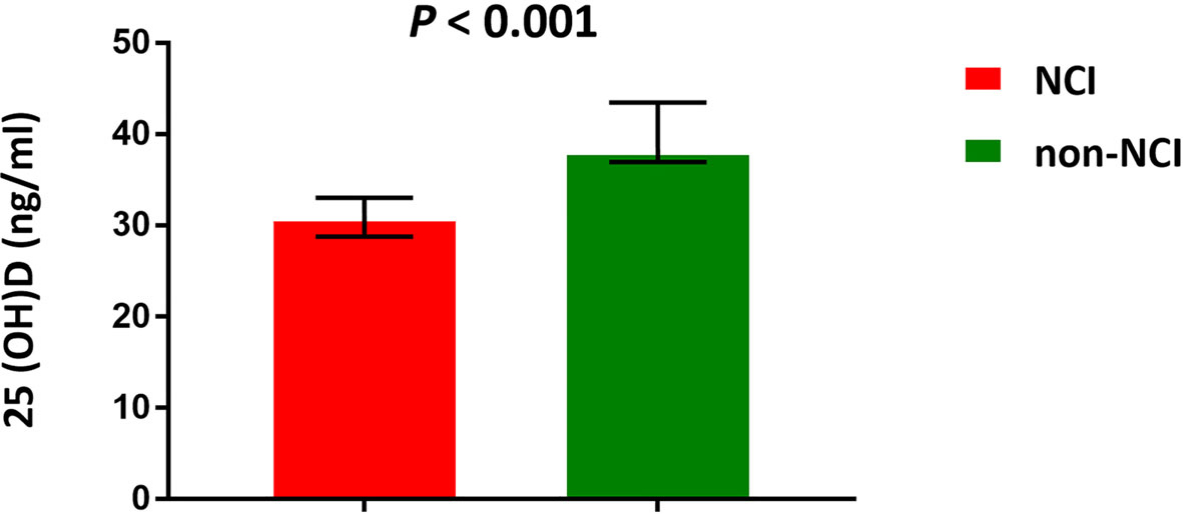
Median concentrations of plasma 25(OH)D. NCI group, HIV-infected persons with neurocognitive impairment; non-NCI, HIV-infected persons without neurocognitive impairment. The error bars represent interquartile range.

We further measured the level of plasma 25(OH)D to verify our previous results. The median 25(OH)D level was 33.1 ng/ml in this population. The plasma 25(OH)D was deficient in one (1%) participant, insufficient in 23 (22.5%) participants, and normal in 78 (76.5%) participants. The 25(OH)D level was significantly lower in the NCI group than that in the non-NCI group (*P* < 0.001) (**Figure 5**). The plasma 25(OH)D was negatively associated with the left (*P* = 0.021) and the right CIMT (*P* = 0.007). Furthermore, the plasma 25(OH)D was positively associated with *Faecalibacterium*, Corprococcus_2, and Ruminococcaceae_NK4A214_groups but negatively associated with Ruminococcus_1, Eubacterium_eligens_group, and uncultured_bacterium (**Table 2**).

**Table 2:** The correlation between 25(OH)D and the abundance of genera [left and right carotid intima-media thickness (CIMT)].

	Correlation	*P*-value	Adjusted *P-*value
Left CIMT	-0.272	0.006^*^	0.021^*^
Right CIMT	-0.319	0.001^**^	0.007^**^
Faecalibacterium	0.217	0.028^*^	0.073
Ruminococcus_1	-0.289	0.003^**^	0.014^*^
Coprococcus_2	0.335	<0.001^***^	0.007^**^
Ruminococcaceae_NK4A214_group	0.188	0.049^*^	0.101
Eubacterium._eligens_group	-0.245	0.013^*^	0.043^*^
uncultured_bacterium	-0.196	0.049^*^	0.099

*P < 0.05; **P < 0.01; ***P < 0.001.

## Discussion

To the best of our knowledge, this is the first study using an integrated 16S rRNA gene sequencing and non-targeted fecal metabolomics approach to investigate the association of the gut microbiota and fecal metabolic phenotype with NCI in HIV‐infected individuals. We found some significant differences between the NCI and non-NCI groups in terms of the abundance of gut microbiota at the genera level as well as in levels of several bacteria-related fecal metabolites. We also found significant correlations between the abundance of microbiota and fecal metabolites.

A number of studies suggested that gut microbiota dysbiosis in HIV‐infected individuals is common (Ribeiro et al., 2017; Bandera et al., 2018). However, the role of gut microbiota dysbiosis in the pathogenesis of NCI in HIV‐infected individuals is still an understudied area. We only identified one study from literature that showed a significantly lower a-diversity in participants with HAND compared to those without HAND in HIV-infected individuals. To exclude confounding bias, the authors matched the two groups for well-known confounders, including age, gender, education status, CD4 count, and sexual preference, that underwent further analysis. They found that there were no significant differences in a-diversity, β-diversity, and microbiota composition between the two groups (Zhang et al., 2019). In our study, the NCI and non-NCI groups also showed comparable a-diversity and β-diversity with comparable potential confounders, while some significant differences in gut composition were identified between the two groups.

Among the genera with observed differences between the NCI and non-NCI groups, *Faecalibacterium*, Corprococcus_2, Ruminococcaceae_NK4A214_group, and Ruminococcus_1 contain butyrate-producing bacteria (BPB). They were increased in the non-NCI group compared with the NCI group and were negatively correlated with CIMT. This result is supported by previously observed links between BPB and numerous neurocognitive disorders, including Parkinson’s disease, Alzheimer’s disease, and depression (Liu et al., 2020; Nuzum et al., 2020). It is now well established that BPB can provide energy for intestinal cells and to protect the gut barrier. The loss of BPB (*Faecalibacterium* or *Coprococcus*) could lead to a loss of integrity of the gut barrier and protection against intestinal epithelium inflammation (Liu et al., 2020; Nuzum et al., 2020). Our findings support that a low abundance of BPB was associated with NCI and may support a link between altered gut microbiota and the chronic, low-grade inflammation often observed in NCI patients. We also found that *Klebsiella*, which is an opportunistic pathogenic taxon, was increased in the NCI group and positively correlated with CIMT. *Klebsiella* has been found to be frequently distributed in hypertensive gut microbiome (Yan et al., 2017). Thus, BPB and *Klebsiella* may play a role in the potential pathogenesis behind NCI and carotid atherosclerosis.

We further studied the metabolic byproducts of intestinal bacteria in fecal samples. NCI appeared to be associated with several fecal metabolites. Consistent with previous studies, we found that specific BAs and bioactive lipids [glycerophospholipids, fatty acids, eicosanoids, and endocannabinoids (anandamide, 20:l, *n* - 9)] may have a role in influencing the cognitive function (MahmoudianDehkordi et al., 2019; Baptista et al., 2020). Increasing evidence suggests that BAs could serve as biomarkers of cognitive aging and Alzheimer’s disease (MahmoudianDehkordi et al., 2019; Nho et al., 2019). Recently, a significant increase in the levels of bacterially produced secondary BAs (deoxycholic acid) and their conjugated forms in AD patients was noted compared to those in cognitively normal older adults (MahmoudianDehkordi et al., 2019). Earlier studies also identified increased levels of several secondary BAs in mild cognitive impairment and AD patients compared with those of cognitively normal controls (Mapstone et al., 2014; Marksteiner et al., 2018). However, inconsistent with those previous findings, we observed increased levels of specific primary BAs (glycocholic acid and chenodeoxycholic acid glycine conjugate) in NCI patients compared to those in non-NCI subjects. The differences may be attributed to the different population. To our best knowledge, most of the previous studies were conducted in the general population. Thus, on the one hand, the links that we observed need to be tested in large populations. Moreover, additional experimental studies are needed to fully define the mechanistic roles of BAs in the development of NCI in HIV-infected patients.

In addition, we, for the first time, identified significant declines of multiple dietary and nutritional constituents known to exert anti-inflammatory and antioxidant properties, including vitamin D, terpenoids, and resolvin D1 in the NCI group compared with the non-NCI group. Terpenoids may be considered useful modulators of brain-derived neurotrophic factor (BDNF) in CNS diseases—for example, *in vitro* studies have found that the BDNF expression activities were positively associated with the terpenoids (Sangiovanni et al., 2017; Gonulalan et al., 2020). For resolving D1, a study reported that the early chronic treatment of rats overexpressing human α-synuclein with resolving D1 prevents central and peripheral inflammation as well as neuronal dysfunction and motor deficits. They also reported that central and peripheral resolving D1 is decreased in early-PD patients (Krashia et al., 2019). However, human evidence is very limited. Further clinical studies are needed to confirm these constituents as diagnostic biomarkers and disease-modifying agents.

Vitamin D may be an important relevant dietary aspect in HIV-infected people for several reasons. First, previous studies reported that vitamin D insufficiency [25(OH)D <30 ng/ml] is highly prevalent, up to 60–85% depending on demographic information, geographic information, and type of cART in HIV-1 infected populations (Manion et al., 2017; Mastaglia et al., 2017). Second, vitamin D insufficiency or deficiency has been associated with NCI, subclinical vascular disease, and several HIV disease outcomes (Vergori et al., 2019). In this study, the median plasma 25(OH)D was 33.1 ng/ml, and the prevalence of vitamin D insufficiency was 22.5%. Our study population had relatively better vitamin D status when compared with the previous data (Manion et al., 2017; Mastaglia et al., 2017), whereas we still found the association between vitamin D insufficiency and NCI. On one hand, vitamin D is the main subclass of fecal metabolites decreased in the NCI group compared with the non-NCI group. On the other hand, the plasma 25(OH)D in the NCI group was significantly lower than that in the non-NCI group. 25(OH)D was also negatively associated with the left and right CIMT. 25(OH)D has been associated to NCI and CIMT in many studies (Vergori et al., 2019; Zugic Soares et al., 2019; Gonzalez-Martin et al., 2020). Different mechanisms have been suggested to explain the neuroprotective effects of 25(OH)D, including the regulation of inflammation, reactive oxygen species, and calcium homeostasis as well as vessel protection (Schlogl and Holick, 2014). It should be noted that we firstly found that fecal vitamin D metabolites were associated with NCI. There is little information in literature on fecal vitamin D level and health risk. To our knowledge, only one recent study found that vitamin D was significantly decreased in the feces of hypertension patients and positively correlated with hypertension‐reduced bacterial genera (Bashir et al., 2016). It should be noted that regulation of the circulating levels of 25(OH)D depends mainly on sun exposure and nutritional intake. As an observational study, it remains unclear whether the decreased level of 25(OH)D in the NCI group (we observed differences) that we note causes or is a consequence of NCI. Therefore, we could not exclude the possibility of an inverse causal link.

In addition, we evaluated the association between gut microbiota perturbations and fecal metabolites. Herein we observed significant associations of BPB and *Klebsiella* with several fecal metabolites, including vitamin D metabolites. The metabolites with a higher level in the NCI group compared to the non-NCI group were negatively correlated with BPB and positively correlated with *Klebsiella*. The metabolites with a lower level in the NCI group were positively correlated with BPB, whereas these were negatively correlated with Klebsiella. These findings suggest that these metabolites may mediate the associations of BPB and *Klebsiella* with NCI. Consistently, both fecal and plasma vitamin D were positively associated with BPB (*Faecalibacterium*, Corprococcus_2, and Ruminococcaceae_NK4A214_groups), while these were negatively associated with Ruminococcus_1. In agreement with our findings, some studies reported an increase in abundance of *Coprococcus* and *Faecalibacterium* and a decreased abundance of genus *Ruminococcus* after vitamin D supplementation (Bashir et al., 2016; Naderpoor et al., 2019). However, open-label pilot studies and randomized, double-blind trials examining the effect of vitamin D on human gut microbiota did not reach a conclusion. Further studies are needed to fully understand the relation between vitamin D and gut microbiota.

The present study has some limitations. First, the relatively small sample size might lead to a low statistical power. Second, the study design cannot prescribe a causal mechanism. Future studies are needed to elucidate the causal relationship.

## Conclusion

BPB, *Klebsiella*, and associated metabolites may play a role in the pathogenesis of NCI. In addition, this is the first time that we found vitamin D, both in feces and blood, to be negatively associated with NCI and positively correlated with BPB.

## Data Availability Statement

The raw data supporting the conclusions of this article will be made available by the authors, without undue reservation.

## Ethics Statement

The studies involving human participants were reviewed and approved by the Committee of the School of Public Health at Fudan University. The patients/participants provided their written informed consent to participate in this study.

## Author Contributions

RD and NH proposed and developed the research question. HL and NH generally supervised the study. RD, XC, RS, SY, JL, BZ, XX, and KW contributed to data collection and data management. WS contributed to laboratory management and tests. RD performed data analysis. RD, NH, and XS wrote, reviewed, and edited the manuscript. DD provided expertise on neuropsychological tests. All authors contributed to the article and approved the submitted version.

## Funding

This study was supported by the National Natural Science Foundation of China (81773485) and the China National Science and Technology Major Projects on Infectious Diseases (2018ZX10721102-004) and partially supported by the Shanghai Municipal Health Commission (GWV-10.1-XK16).

## Conflict of Interest

The authors declare that the research was conducted in the absence of any commercial or financial relationships that could be construed as a potential conflict of interest.

## Publisher’s Note

All claims expressed in this article are solely those of the authors and do not necessarily represent those of their affiliated organizations, or those of the publisher, the editors and the reviewers. Any product that may be evaluated in this article, or claim that may be made by its manufacturer, is not guaranteed or endorsed by the publisher.
