# Supplementary material for: Gut Microbiota and Fecal Metabolites Associated With Neurocognitive Impairment in HIV-Infected Population
Source: Front Cell Infect Microbiol. 2021 Oct 25;11:723840. doi: 10.3389/fcimb.2021.723840 (PMC8574817; doi:10.3389/fcimb.2021.723840)
Supplement: Supplementary file 1 [file Table_1.docx]

Supplementary Material

Supplementary Methods

**Neuropsychological tests**

Multiple cognitive–motor ability domains that contently found in HIV-associated brain disease in patients from the United States were tapped in the test battery. The battery was carefully reviewed and approved to be culturally appropriate for the study populations in China by mental health professionals [1]. Ten NP tests were used to evaluate seven domains: semantic verbal fluency (animals); Hopkins Verbal Learning Test; Brief Visuospatial Memory Test; Stroop Color and Word Test; Trail Making Test (Parts A and B); Wisconsin Card Sorting Test 64 Card Version (WCST-64); Grooved Pegboard Test; Paced Auditory Serial Addition Test (PASAT); the Digit Symbol, Symbol Search, and Letter-Number Sequencing tests from the Wechsler Adult Intelligence Scale (WAIS-III).

Scaled scores were converted from raw test scores from these tests. For each test, standardized T scores were generated with the mean of 50 (standard deviation, 10) and adjusted for age, sex, and education. Based on T scored, deficit scores (DSs) ranging from 0 (no deficit) to 5 (severe deficit) were created: T score ≥40 = DS score of 0; T score 35–39 = DS score of 1; T score 30–34 = DS score of 2; T score 25–29 = DS score of 3; T score 20–24 = DS of 4; T score <20 = DS score of 5. To generate global T and global deficit scores (GDS), domain T and DS took the average scores of individual tests within each domain and across all tests respectively. The global cognitive score was defined by the global deficit scores (GDS) dichotomized as impaired (GDS ≥ 0.5) or unimpaired (GDS < 0.5).

**16S rRNA sequencing**

***DNA extraction***

Total genomic DNA was extracted using DNA Extraction Kit following the manufacturer’s instructions. Quality and quantity of DNA was verified with NanoDrop and agarose gel. Extracted DNA was diluted to a concentration of 1 ng/μl and stored at -20 °C until further processing. The diluted DNA was used as template for PCR amplification of bacterial 16S rRNA genes with the barcoded primers and Takara Ex Taq (Takara). For bacterial diversity analysis, V3-V4 (or V4-V5) variable regions of 16S rRNA genes was amplified with universal primers 343 F and 798 R (or 515F and 907R for V4-V5 regions).

***Library Construction***

Amplicon quality was visualized using gel electrophoresis, purified with AMPure XP beads (Agencourt), and amplified for another round of PCR. After purified with the AMPure XP beads again, the final amplicon was quantified using Qubit dsDNA assay kit. Equal amounts of purified amplicon were pooled for subsequent sequencing.

***Data Processing and Quality Control***

Raw sequencing data were in FASTQ format. Paired-end reads were then preprocessed using Trimmomatic software to detect and cut off ambiguous bases (N). It also cut off low quality sequences with average quality score below 20 using sliding window trimming approach [2]. After trimming, paired-end reads were assembled using FLASH software [3]. Parameters of assembly were: 10bp of minimal overlapping, 200bp of maximum overlapping and 20 % of maximum mismatch rate. Sequences were performed further denoising as follows: reads with ambiguous, homologous sequences or below 200 bp were abandoned. Reads with 75 % of bases above Q20 were retained. Then, reads with chimera were detected and removed. These two steps were achieved using QIIME software (version 1.8.0) [4]. Clean reads were subjected to primer sequences removal and clustering to generate operational taxonomic units (OTUs) using Vsearch software with 97 % similarity cutoff. The representative read of each OTU was selected using QIIME package. All representative reads were annotated and blasted against Silva database Version 123 (or Greengens) (16s/18s rDNA) using RDP classifier (confidence threshold was 70 %) [5]. All representative reads were annotated and blasted against Unite database (ITSs rDNA) using blast, followed by alpha and beta diversity analyses. Accumulation curve is presented in [Figure S1](https://www.ncbi.nlm.nih.gov/pmc/articles/PMC7520699/figure/fig2/). The curves of each sample tended to flatten, suggesting that increasing sequencing depths did not help to discover new OTUs. The valid_tags, valid_percent, valid minLength, valid meanLength, valid maxLength, subsample_depth, OTU_counts and total_OTUs for each sample are shown in Table S1. Figure S2 showed the distribution of clean tags.


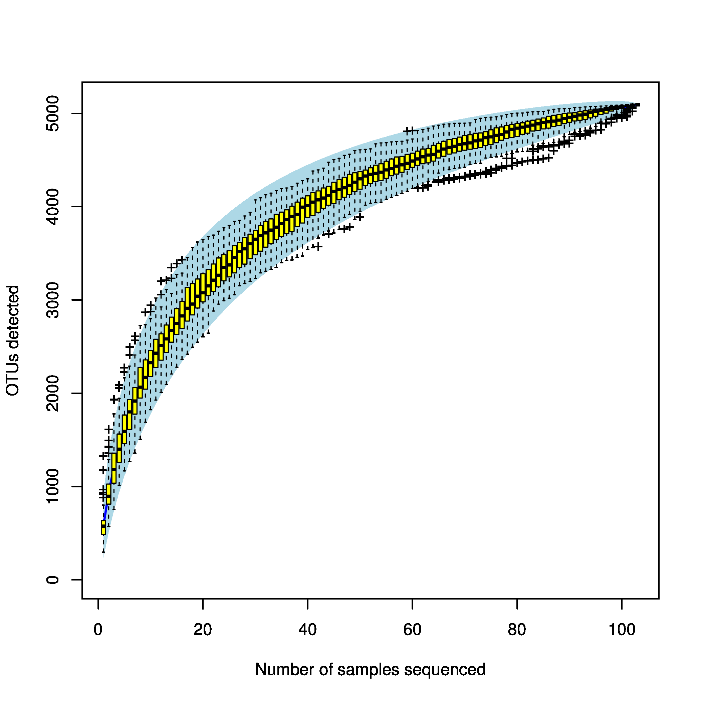


Figure S1. Accumulation curves for each subgroup. The x-axis is the number of samples, and the y-axis is the number of operational taxonomic units (OTUs) detected.


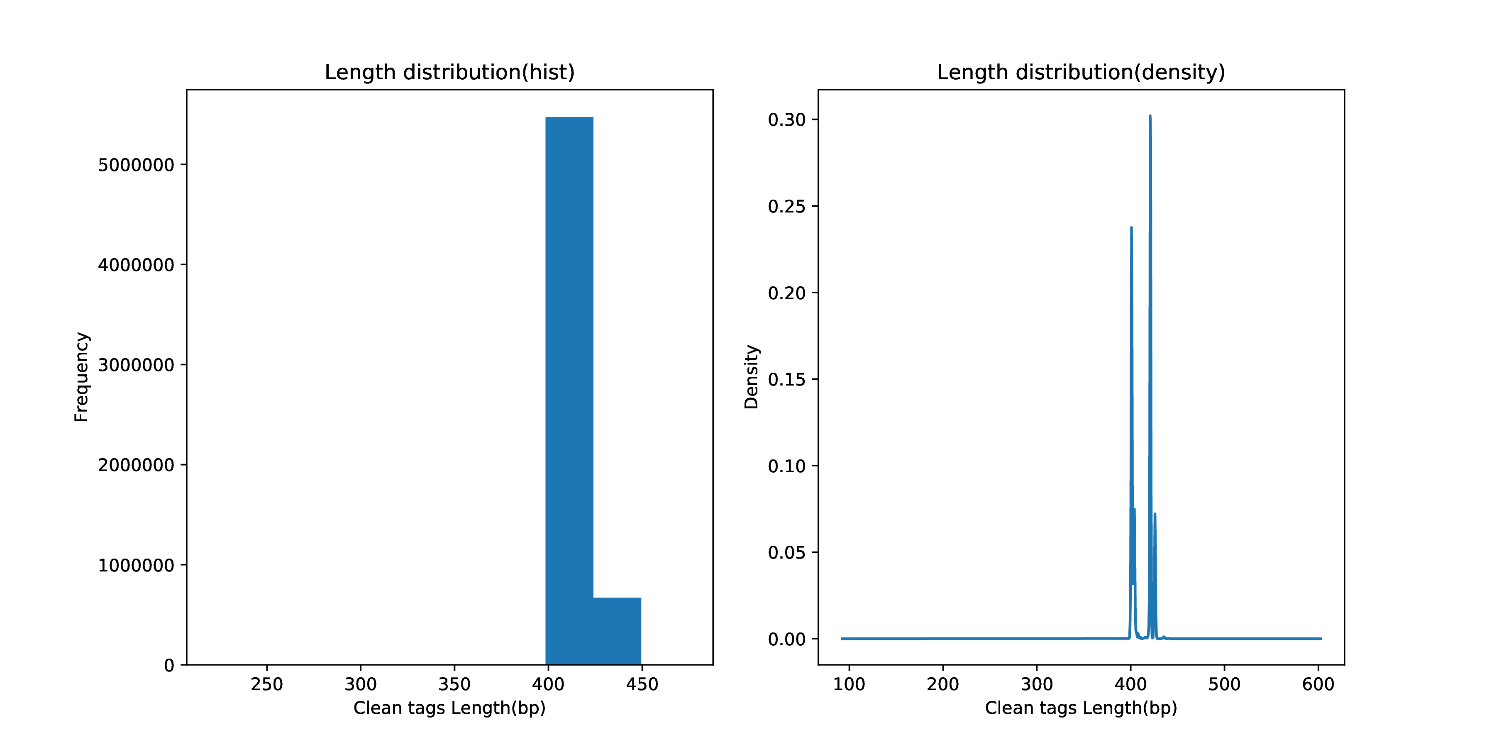


Figure S2. Distribution of Clean tags. Histogram, the x-axis is the length of tags, and the y-axis is the number of tags; density figure, the x-axis is the length of tags, and the y-axis is the probability density.

Table S1. The distribution of tags for each sample

| Sample_ID | clean_tags | valid_tags | valid_percent | valid minLength | valid meanLength | valid maxLength | subsample_depth | OTU_counts | Total_OTUs |
| --- | --- | --- | --- | --- | --- | --- | --- | --- | --- |

| A793 | 56301 | 52480 | 93.21% | 248 | 412.45 | 441 | 23816 | 829 | 5093 |
| --- | --- | --- | --- | --- | --- | --- | --- | --- | --- |
| A790 | 54974 | 52127 | 94.82% | 229 | 412.18 | 449 | 23816 | 971 | 5093 |
| A786 | 49930 | 45460 | 91.05% | 259 | 411.49 | 441 | 23816 | 759 | 5093 |
| A7118 | 33402 | 29771 | 89.13% | 313 | 413.95 | 440 | 23816 | 483 | 5093 |
| A457 | 61227 | 58241 | 95.12% | 236 | 412.89 | 441 | 23816 | 575 | 5093 |
| A09 | 58200 | 54715 | 94.01% | 256 | 412.87 | 441 | 23816 | 498 | 5093 |
| A012 | 59476 | 54382 | 91.44% | 228 | 412.77 | 441 | 23816 | 643 | 5093 |
| A098 | 56086 | 51234 | 91.35% | 229 | 410.35 | 440 | 23816 | 752 | 5093 |
| A010 | 61969 | 59155 | 95.46% | 220 | 407.21 | 441 | 23816 | 1005 | 5093 |
| A01 | 58169 | 55519 | 95.44% | 256 | 418.43 | 449 | 23816 | 463 | 5093 |
| A013 | 61041 | 56731 | 92.94% | 259 | 408.85 | 441 | 23816 | 264 | 5093 |
| A011 | 61813 | 54430 | 88.06% | 229 | 413.32 | 442 | 23816 | 446 | 5093 |
| A1102 | 59642 | 56540 | 94.80% | 258 | 412.1 | 440 | 23816 | 673 | 5093 |
| A1103 | 58184 | 54032 | 92.86% | 258 | 417.53 | 453 | 23816 | 524 | 5093 |
| A115 | 59124 | 52828 | 89.35% | 259 | 416.09 | 441 | 23816 | 297 | 5093 |
| A119 | 60477 | 56182 | 92.90% | 259 | 414.81 | 449 | 23816 | 402 | 5093 |
| A667 | 59721 | 54956 | 92.02% | 258 | 416.15 | 442 | 23816 | 458 | 5093 |
| A671 | 60911 | 57209 | 93.92% | 259 | 416.11 | 449 | 23816 | 554 | 5093 |
| A6110 | 61134 | 57594 | 94.21% | 256 | 410.27 | 442 | 23816 | 625 | 5093 |
| A2108 | 61209 | 58225 | 95.12% | 229 | 416.51 | 440 | 23816 | 430 | 5093 |
| A235 | 60504 | 55770 | 92.18% | 251 | 413.51 | 439 | 23816 | 585 | 5093 |
| A228 | 61764 | 55307 | 89.55% | 259 | 409.79 | 441 | 23816 | 522 | 5093 |
| A222 | 58288 | 53794 | 92.29% | 258 | 406.32 | 440 | 23816 | 512 | 5093 |
| A242 | 59714 | 57024 | 95.50% | 229 | 417.03 | 449 | 23816 | 342 | 5093 |
| A560 | 59917 | 56803 | 94.80% | 229 | 420.83 | 451 | 23816 | 374 | 5093 |
| A679 | 59185 | 54459 | 92.01% | 250 | 413.51 | 441 | 23816 | 801 | 5093 |
| A0100 | 58895 | 52970 | 89.94% | 229 | 412.46 | 441 | 23816 | 611 | 5093 |
| A352 | 60333 | 55093 | 91.31% | 259 | 414.58 | 441 | 23816 | 435 | 5093 |
| A355 | 60269 | 53495 | 88.76% | 258 | 410.48 | 442 | 23816 | 609 | 5093 |
| A344 | 59356 | 54001 | 90.98% | 232 | 412.56 | 443 | 23816 | 920 | 5093 |
| A349 | 60716 | 52780 | 86.93% | 229 | 413.98 | 441 | 23816 | 567 | 5093 |
| B794 | 59957 | 55292 | 92.22% | 256 | 408.96 | 441 | 23816 | 613 | 5093 |
| B787 | 58633 | 54062 | 92.20% | 229 | 409.71 | 441 | 23816 | 694 | 5093 |
| B7121 | 60975 | 56944 | 93.39% | 259 | 410.53 | 440 | 23816 | 504 | 5093 |
| B458 | 61514 | 55968 | 90.98% | 220 | 415.81 | 449 | 23816 | 1331 | 5093 |
| B099 | 61007 | 55158 | 90.41% | 220 | 412.32 | 441 | 23816 | 580 | 5093 |
| B05 | 60856 | 57662 | 94.75% | 256 | 412.74 | 453 | 23816 | 941 | 5093 |
| B02 | 60117 | 56667 | 94.26% | 229 | 419.32 | 440 | 23816 | 449 | 5093 |
| B06 | 61552 | 55248 | 89.76% | 229 | 410.53 | 442 | 23816 | 568 | 5093 |
| B08 | 58791 | 52436 | 89.19% | 256 | 416.62 | 441 | 23816 | 537 | 5093 |
| B03 | 61485 | 55913 | 90.94% | 243 | 408.12 | 441 | 23816 | 679 | 5093 |
| B07 | 61314 | 56531 | 92.20% | 258 | 405.49 | 442 | 23816 | 418 | 5093 |
| B118 | 58332 | 53939 | 92.47% | 259 | 408.96 | 441 | 23816 | 604 | 5093 |
| B1101 | 59626 | 56807 | 95.27% | 220 | 410.41 | 441 | 23816 | 689 | 5093 |
| B114 | 61619 | 54497 | 88.44% | 259 | 414.48 | 441 | 23816 | 497 | 5093 |
| B6112 | 59467 | 53494 | 89.96% | 259 | 413.54 | 441 | 23816 | 426 | 5093 |
| B670 | 60634 | 57843 | 95.40% | 256 | 418.97 | 452 | 23816 | 885 | 5093 |
| B674 | 59170 | 54360 | 91.87% | 259 | 408.62 | 441 | 23816 | 426 | 5093 |
| B675 | 61429 | 56606 | 92.15% | 256 | 409.83 | 442 | 23816 | 711 | 5093 |
| B668 | 61899 | 58578 | 94.63% | 259 | 414.91 | 442 | 23816 | 786 | 5093 |
| B6114 | 58182 | 53194 | 91.43% | 229 | 412.78 | 442 | 23816 | 400 | 5093 |
| B233 | 61042 | 55271 | 90.55% | 256 | 415.42 | 442 | 23816 | 475 | 5093 |
| B223 | 60530 | 54868 | 90.65% | 228 | 411.65 | 441 | 23816 | 453 | 5093 |
| B229 | 61886 | 57712 | 93.26% | 259 | 411.62 | 442 | 23816 | 661 | 5093 |
| B238 | 60733 | 56632 | 93.25% | 228 | 408.34 | 441 | 23816 | 630 | 5093 |
| B2106 | 60538 | 55473 | 91.63% | 220 | 415.41 | 439 | 23816 | 455 | 5093 |
| B234 | 59171 | 54440 | 92.00% | 259 | 406.26 | 441 | 23816 | 533 | 5093 |
| B224 | 58949 | 55536 | 94.21% | 258 | 408.63 | 441 | 23816 | 453 | 5093 |
| B237 | 58367 | 52360 | 89.71% | 259 | 415.12 | 441 | 23816 | 621 | 5093 |
| B2105 | 58368 | 52008 | 89.10% | 256 | 410.16 | 440 | 23816 | 608 | 5093 |
| B566 | 59492 | 55308 | 92.97% | 229 | 418.76 | 441 | 23816 | 542 | 5093 |
| B565 | 60884 | 55020 | 90.37% | 251 | 412.1 | 441 | 23816 | 491 | 5093 |
| B561 | 58465 | 46432 | 79.42% | 228 | 415.31 | 441 | 23816 | 430 | 5093 |
| B350 | 59097 | 55146 | 93.31% | 236 | 406.2 | 448 | 23816 | 719 | 5093 |
| B354 | 61280 | 58073 | 94.77% | 259 | 407.66 | 442 | 23816 | 652 | 5093 |
| B348 | 58108 | 53201 | 91.56% | 228 | 413.12 | 441 | 23816 | 590 | 5093 |
| B353 | 59873 | 55017 | 91.89% | 259 | 408.45 | 441 | 23816 | 661 | 5093 |
| B346 | 60481 | 52619 | 87.00% | 229 | 410.77 | 441 | 23816 | 664 | 5093 |
| C110 | 61135 | 58541 | 95.76% | 258 | 421.05 | 442 | 23816 | 576 | 5093 |
| C151 | 60213 | 49284 | 81.85% | 256 | 412.97 | 449 | 23816 | 1181 | 5093 |
| C152 | 61366 | 56359 | 91.84% | 258 | 408.33 | 440 | 23816 | 502 | 5093 |
| C153 | 59987 | 55065 | 91.79% | 259 | 407 | 441 | 23816 | 615 | 5093 |
| C154 | 61812 | 56312 | 91.10% | 229 | 415.37 | 441 | 23816 | 490 | 5093 |
| C746 | 59968 | 53175 | 88.67% | 229 | 409.08 | 442 | 23816 | 635 | 5093 |
| C747 | 60676 | 53238 | 87.74% | 259 | 413.7 | 441 | 23816 | 516 | 5093 |
| C748 | 61224 | 54684 | 89.32% | 256 | 407.98 | 441 | 23816 | 594 | 5093 |
| C763 | 58866 | 50979 | 86.60% | 259 | 411.01 | 440 | 23816 | 636 | 5093 |
| C01 | 61639 | 54384 | 88.23% | 258 | 411.47 | 441 | 23816 | 405 | 5093 |
| C03 | 61588 | 55848 | 90.68% | 258 | 412.57 | 440 | 23816 | 370 | 5093 |
| C04 | 59222 | 56508 | 95.42% | 256 | 409.9 | 441 | 23816 | 688 | 5093 |
| C429 | 60707 | 55260 | 91.03% | 258 | 414.62 | 441 | 23816 | 453 | 5093 |
| C431 | 60005 | 46903 | 78.17% | 258 | 409.49 | 439 | 23816 | 478 | 5093 |
| C635 | 58264 | 46734 | 80.21% | 229 | 410.28 | 441 | 23816 | 402 | 5093 |
| C639 | 60509 | 47422 | 78.37% | 258 | 411.73 | 441 | 23816 | 542 | 5093 |
| C661 | 61790 | 54957 | 88.94% | 259 | 416.05 | 442 | 23816 | 620 | 5093 |
| C641 | 59068 | 55559 | 94.06% | 258 | 410.47 | 441 | 23816 | 418 | 5093 |
| C644 | 60445 | 53834 | 89.06% | 220 | 412.55 | 441 | 23816 | 657 | 5093 |
| C645 | 52446 | 41639 | 79.39% | 258 | 408.69 | 441 | 23816 | 687 | 5093 |
| C213 | 61638 | 42774 | 69.40% | 259 | 415.38 | 441 | 23816 | 512 | 5093 |
| C217 | 61948 | 49274 | 79.54% | 259 | 415.06 | 441 | 23816 | 574 | 5093 |
| C219 | 61700 | 54412 | 88.19% | 229 | 414.04 | 441 | 23816 | 585 | 5093 |
| C220 | 59608 | 53264 | 89.36% | 259 | 411.3 | 440 | 23816 | 682 | 5093 |
| C221 | 61013 | 55482 | 90.93% | 258 | 413.41 | 441 | 23816 | 435 | 5093 |
| C222 | 61398 | 57858 | 94.23% | 250 | 416.58 | 441 | 23816 | 366 | 5093 |
| C224 | 60504 | 56749 | 93.79% | 251 | 416.03 | 441 | 23816 | 659 | 5093 |
| C255 | 61786 | 36958 | 59.82% | 259 | 411.1 | 441 | 23816 | 738 | 5093 |
| C215 | 59103 | 42204 | 71.41% | 259 | 411.92 | 441 | 23816 | 536 | 5093 |
| C532 | 61934 | 55113 | 88.99% | 258 | 412.25 | 442 | 23816 | 463 | 5093 |
| C533 | 60171 | 57014 | 94.75% | 258 | 403.12 | 441 | 23816 | 523 | 5093 |
| C534 | 60247 | 54345 | 90.20% | 259 | 413.12 | 440 | 23816 | 620 | 5093 |
| C558 | 60611 | 50392 | 83.14% | 259 | 414.24 | 441 | 23816 | 562 | 5093 |
| C559 | 60623 | 51328 | 84.67% | 259 | 420.44 | 442 | 23816 | 424 | 5093 |

Reference:

1. Cysique, L.A., et al., Neurobehavioral effects of HIV-1 infection in China and the United States: a pilot study. J Int Neuropsychol Soc, 2007. 13(5): p. 781-90.
2. Bolger AM, Lohse M and Usadel B. Trimmomatic: a flexible trimmer for Illumina sequence data. Bioinformatics, 2014, 30(15): 2114-2120.
3. Magoč T, Salzberg SL. FLASH: fast length adjustment of short reads to improve genome assemblies. Bioinformatics. 2011;27(21):2957-2963. doi:10.1093/bioinformatics/btr507.i
4. Caporaso J G, Kuczynski J, Stombaugh J, et al. QIIME allows analysis of high-throughput community sequencing data. Nature Methods, 2010, 7(5): 335–336.
5. Edgar RC, Haas BJ, Clemente JC, et al., UCHIME improves sensitivity and speed of chimera detection. Bioinformatics, 2011, 27(16): 2194-2200.

**Measurement of faecal Metabolomics**

***Chemicals***

All chemicals and solvents were analytical for HPLC grade. Water, methanol, acetonitrile, formic acid were purchased from CNW Technologies GmbH (Düsseldorf, Germany). L-2 chlorophenylalanine was from Shanghai Hengchuang Bio-technology Co., Ltd. (Shanghai, China).

***Sample Preparation***

The sample was transferred to a 1.5-mL Eppendorf tube. Two small steel balls were added to the tube. 20 μL internal standard (2-chloro-l-phenylalanine in methanol, 0.3 mg/mL) and extraction solvent with methanol /water (4/1, v/v) were added to each sample. Samples were stored at -20 °C for 5 min and then grinded at 60 HZ for 2 min, ultrasonicated at ambient temperature (25 °C to 28 °C) for 10 min, stored at -20 °C for 30 min. The extract was centrifuged at 13000 rpm, 4 °C for 15 min. The supernatant in a glass vial was dried in a freeze concentration centrifugal dryer. The mixture of methanol and water (1/4, vol/vol) were added to each sample, samples vortexed for 30 s, then placed at 4°C for 2 min. Samples were centrifuged at 13000 rpm, 4 °C for 5 min. The supernatants from each tube were collected using crystal syringes, filtered through 0.22 μm microfilters and transferred to LC vials. The vials were stored at 4°C until LC -MS analysis. QC samples were prepared by mixing aliquots of the all samples to be a pooled sample.

***Data Preprocessing and Statistical Analysis***

The acquired LC-MS raw data were analyzed by the progenesis QI software (Waters Corporation，Milford, USA) using the following parameters. Precursor tolerance was set 5 ppm, fragment tolerance was set 10 ppm, and retention time (RT) tolerance was set 0.02 min. Internal standard detection parameters were deselected for peak RT alignment, isotopic peaks were excluded for analysis, and noise elimination level was set at 10.00, minimum intensity was set to 15 % of base peak intensity. The Excel file was obtained with three-dimension data sets including m/z, peak RT and peak intensities, and RT–m/z pairs were used as the identifier for each ion. The resulting matrix was further reduced by removing any peaks with missing value (ion intensity = 0) in more than 50 % samples. The internal standard was used for data QC (reproducibility).

Metabolites were identified by progenesis QI (Waters Corporation, Milford, USA) Data Processing Software, based on public databases such as http://www.hmdb.ca/; http://www.lipidmaps.org/ and self-built databases. The positive and negative data were combined to get a combine data which was imported into R ropls package. Principle component analysis (PCA) and (orthogonal) partial least-squares-discriminant analysis (O)PLS-DA were carried out to visualize the metabolic alterations among experimental groups, after mean centering (Ctr) and Pareto variance (Par) scaling, respectively. The Hotelling’s T2 region, shown as an ellipse in score plots of the models, defines the 95 % confidence interval of the modeled variation. Variable importance in the projection (VIP) ranks the overall contribution of each variable to the OPLS-DA model, and those variables with VIP > 1 are considered relevant for group discrimination.

In this study, the default 7-round cross-validation was applied with 1/seventh of the samples being excluded from the mathematical model in each round, in order to guard against overfitting. The differential metabolites were selected on the basis of the combination of a statistically significant threshold of variable influence on projection (VIP) values obtained from the OPLS- DA model and *p* values from a two-tailed Student’s t test on the normalized peak areas, where metabolites with VIP values larger than 1.0 and *p* values less than 0.05 were considered as differential metabolites.

## Supplementary Results

**Table S2. Characteristics of the non-NCI group**

| **Characteristics** | **Non-NCI group (n=122) ^a^** | **Non-NCI group (n=35) ^b^** | ***P* values** |
| --- | --- | --- | --- |
| **Demographics** |  |  |  |
| Sex, n (%) |  |  |  |
| Male | 97 (79.5) | 25 (71.4) | 0.844 |
| Female | 25 (20.5) | 10 (29.4) |  |
| Age, years, (mean ± SD) | 56.0 ± 9.6 | 55.3 ± 9.3 | 0.635 |
| BMI, kg/m^2^, (mean ± SD) | 22.3 ± 3.4 | 22.9 ± 2.6 | 0.964 |
| Education, n (%) |  |  |  |
| ≤ Primary school | 52 (42.6) | 20 (49.0) | 0.344 |
| middle school | 46 (37.7) | 11 (31.4) |  |
| ≥ High school | 24 (19.7) | 4 (8.6) |  |
| Current smoker, n (%) |  |  |  |
| Yes | 34 (27.9 %) | 13 (37.1 %) | 0.269 |
| No | 88 (72.1 %) | 22 (62.9 %) |  |
| Current alcohol use, n (%) |  |  |  |
| Yes | 36 (29.5 %) | 14 (40.0 %) | 0.497 |
| No | 86 (70.5 %) | 21 (60.0 %) |  |
| **HIV-related characteristics** |  |  |  |
| Time since HIV diagnosis, years, (median, IQR) | 5.0 (3.0, 7.5) | 4.8 (3.2, 7.5) | 0.796 |
| Duration on cART, years, (median, IQR) | 4.9 (4.5, 5.7) | 4.0 (2.9, 7.6) | 0.096 |
| Current CD4 count, cells/μL, (mean ± SD) | 449.3 ± 218.4 | 472.2 ± 230.5 | 0.109 |
| **Laboratory biomarkers** |  |  |  |
| HDL (median, IQR) | 1.1 (0.8, 1.4) | 1.2 (1.0, 1.5) | 0.627 |
| LDL (median, IQR)  CHOL (median, IQR) | 2.5 (1.9, 2.9)  4.9 (4.5, 5.7) | 2.5 (2.1, 3.0)  4.5 (3.9, 4.9) | 0.868  0.357 |
| TG (median, IQR) | 2.0 (1.3, 2.9) | 1.7 (1.0, 3.0) | 0.219 |

^a^ Full sample; ^b^ Final sample; NCI: neurocognitive impairment; BMI: body mass index; IQR: interquartile range; SD: standard deviation;

**Table S3. Characteristics of the NCI group**

| **Characteristics** | **NCI group (n=122) ^a^** | **NCI group**  **(n=67) ^b^** | ***P* values** |
| --- | --- | --- | --- |
| **Demographics** |  |  |  |
| Sex, n (%) |  |  |  |
| Male | 97 (79.5) | 55 (82.1) | 0.987 |
| Female | 25 (20.5) | 12 (17.9) |  |
| Age, years, (mean ± SD) | 56.4 ± 10.3 | 54.6 ± 9.7 | 0.708 |
| BMI, kg/m^2^, (mean ± SD) | 22.3 ± 2.8 | 23.2 ± 3.0 | 0.568 |
| Education, n (%) |  |  |  |
| ≤ Primary school | 75（61.5） | 49 (70.0) | 0.297 |
| middle school | 35（28.7） | 13 (19.4) |  |
| ≥ High school | 12（9.8） | 5 (7.5) |  |
| Current smoker, n (%) |  |  |  |
| Yes | 32（26.2） | 35 (52.2 %) | 0.068 |
| No | 90（73.8） | 32 (47.8 %) |  |
| Current alcohol use, n (%) |  |  |  |
| Yes | 31 (25.4 %) | 26 (38.8 %) | 0.467 |
| No | 91（74.6） | 41 (61.2 %) |  |
| **HIV-related characteristics** |  |  |  |
| Time since HIV diagnosis, years, (median, IQR) | 5.3 (3.5, 7.9) | 5.2 (3.8, 8.2) | 0.682 |
| Duration on cART, years, (median, IQR) | 4.5 (3.0, 6.0) | 4.3 (3.7, 6.3) | 0.778 |
| Current CD4 count, cells/μL, (mean ± SD) | 400.9 ± 220.1 | 414.9 ± 194.9 | 0.823 |
| **Laboratory biomarkers** |  |  |  |
| HDL (median, IQR) | 1.1 (0.9, 1.3) | 1.2 (0.9, 1.5) | 0.366 |
| LDL (median, IQR)  CHOL (median, IQR) | 2.4 (1.9, 3.1)  4.8 (4.1, 5.7) | 2.3 (1.8, 2.9)  4.7 (3.9, 5.4) | 0.956  0.748 |
| TG (median, IQR) | 1.8 (1.2, 2.7) | 2.0 (1.2, 3.2) | 0.160 |

^a^ Full sample; ^b^ Final sample; NCI: neurocognitive impairment; BMI: body mass index; IQR: interquartile range; SD: standard deviation;

**Table S4 Altered gut microbiota compositions in the NCI-group.**

| **Altered microbiota** | ***P* value** | **Adjusted *P* value** |
| --- | --- | --- |
| Coprococcus_2 ↓ | ***<0.001****** | ***<0.001****** |
| Treponema_2 ↓ | ***<0.001****** | ***<0.001****** |
| Rikenellaceae_RC9_gut_group ↓ | ***0.001***** | 0.086 |
| Klebsiella ↑ | ***0·004***** | 0.260 |
| Prevotellaceae_Ga6A1_group ↑ | ***0·009***** | 0.353 |
| Streptococcus ↑ | ***0.015**** | 0.350 |
| Metagenome ↓ | ***0.016**** | 0.354 |
| [Eubacterium]_eligens_group ↑ | ***0.018**** | 0.355 |
| uncultured_bacterium ↑ | ***0.019**** | 0.352 |
| Turicibacter ↑ | ***0.019**** | 0.352 |
| Collinsella ↑ | ***0.025**** | 0.371 |
| Helicobacter ↑ | ***0.025**** | 0.371 |
| Alloprevotella ↑ | ***0.027**** | 0.379 |
| Ruminococcus_1 ↓ | ***0.031**** | 0.396 |
| CAG-56 ↓ | ***0.032**** | 0.396 |
| Faecalibacterium ↓ | ***0.038**** | 0.413 |
| Succinivibrio ↓ | ***0.039**** | 0.412 |
| Catenibacterium ↑ | ***0.039**** | 0.413 |
| Ambiguous_taxa ↑ | ***0.046**** | 0.434 |
| Ruminococcaceae_NK4A214_group ↓ | ***0.046**** | 0.435 |

↑: up-regulated; ↓: down-regulated; Bold italic ^*^*P* < 0.05; Bold italic ^**^*P* < 0.01;

Bold italic ^***^*P* < 0.001. The model was adjusted with sex, age, CD4 count and sexual preference and BMI.

**Table S5. The correlation between the abundance of genera with CD4 count, left and right CIMT.**

|  | **Genera** | **Correlation** | ***P* value** | **Adjusted *P* value** |
| --- | --- | --- | --- | --- |
| CD4 count | Faecalibacterium | 0.007 | 0.943 | 0.970 |
|  | Succinivibrio | 0.080 | 0.432 | 0.910 |
|  | Collinsella | -0.068 | 0.500 | 0.713 |
|  | [Eubacterium]_eligens_group | 0.042 | 0.678 | 0.920 |
|  | Klebsiella | 0.155 | 0.126 | 0.642 |
|  | uncultured_bacterium | -0.116 | 0.251 | 0.588 |
|  | Catenibacterium | 0.034 | 0.740 | 0.886 |
|  | Alloprevotella | 0.084 | 0.408 | 0.647 |
|  | Ruminococcus_1 | 0.002 | 0.985 | 0.985 |
|  | Coprococcus_2 | 0.020 | 0.849 | 0.952 |
|  | Streptococcus | -0.245 | ***0.014^*^*** | 0.872 |
|  | Turicibacter | -0.115 | 0.257 | 0.741 |
|  | metagenome | -0.138 | 0.173 | 0.806 |
|  | Prevotellaceae_Ga6A1_group | -0.124 | 0.223 | 0.773 |
|  | Rikenellaceae_RC9_gut_group | -0.152 | 0.134 | 0.340 |
|  | Ambiguous_taxa | -0.012 | 0.910 | 0.969 |
|  | CAG-56 | -0.040 | 0.693 | 0.839 |
|  | Helicobacter | -0.106 | 0.299 | 0.529 |
|  | Ruminococcaceae_NK4A214_group | -0.078 | 0.443 | 0.796 |
|  | Treponema_2 | -0.234 | ***0.020^*^*** | 0.077 |
|  |  |  |  |  |
| Left CIMT | Faecalibacterium | -0.248 | ***0.013^*^*** | 0.341 |
|  | Succinivibrio | 0.022 | 0.826 | 0.997 |
|  | Collinsella | -0.010 | 0.919 | 0.962 |
|  | [Eubacterium]_eligens_group | -0.198 | 0.050 | 0.725 |
|  | Klebsiella | 0.288 | ***0.004^**^*** | 0.301 |
|  | Catenibacterium | 0.047 | 0.643 | 0.886 |
|  | Alloprevotella | 0.043 | 0.675 | 0.819 |
|  | Ruminococcus_1 | -0.232 | ***0.021^*^*** | 0.166 |
|  | Coprococcus_2 | -0.230 | ***0.022^*^*** | 0.574 |
|  | Streptococcus | -0.043 | 0.676 | 0.917 |
|  | Turicibacter | 0.061 | 0.551 | 0.887 |
|  | metagenome | 0.014 | 0.887 | 0.983 |
|  | Prevotellaceae_Ga6A1_group | 0.032 | 0.751 | 0.951 |
|  | Rikenellaceae_RC9_gut_group | 0.113 | 0.268 | 0.463 |
|  | Ambiguous_taxa | -0.116 | 0.254 | 0.814 |
|  | CAG-56 | -0.173 | 0·089 | 0.616 |
|  | Helicobacter | 0.080 | 0.435 | 0.661 |
|  | Ruminococcaceae_NK4A214_group | -0.257 | ***0.010^*^*** | 0.608 |
|  | Treponema_2 | 0.095 | 0.351 | 0.539 |
|  |  |  |  |  |
| Right CIMT | Faecalibacterium | -0.239 | ***0.018^*^*** | 0.341 |
|  | Succinivibrio | -0.020 | 0.843 | 0.996 |
| **Table S5. The correlation between the abundance of genera with CD4 count, left and right CIMT (continued).** | | | | |
|  | Collinsella | 0.113 | 0.269 | 0.487 |
|  | [Eubacterium]_eligens_group | -0.117 | 0.252 | 0.736 |
|  | Klebsiella | 0.237 | ***0.018*^*^** | 0.352 |
|  | uncultured_bacterium | 0.107 | 0.292 | 0.588 |
|  | Catenibacterium | 0.123 | 0.228 | 0.886 |
|  | Alloprevotella | 0.096 | 0.347 | 0.628 |
|  | Ruminococcus_1 | -0.127 | 0.213 | 0.392 |
|  | Coprococcus_2 | -0.187 | 0.065 | 0.952 |
|  | Streptococcus | -0.057 | 0.577 | 0.917 |
|  | Turicibacter | 0.122 | 0.233 | 0.738 |
|  | metagenome | 0.069 | 0.502 | 0.819 |
|  | Prevotellaceae_Ga6A1_group | 0.066 | 0.515 | 0.863 |
|  | Rikenellaceae_RC9_gut_group | 0.132 | 0.194 | 0.418 |
|  | Ambiguous_taxa | -0.010 | 0.918 | 0.969 |
|  | CAG-56 | -0.186 | 0.066 | 0.564 |
|  | Helicobacter | 0·112 | 0.270 | 0.514 |
|  | Ruminococcaceae_NK4A214_group | -0.195 | 0.054 | 0.608 |
|  | Treponema_2 | 0.155 | 0.127 | 0.269 |

Bold italic ^*^*P* < 0.05;

**Table S6. Potential faecal biomarkers and their variation trends in the NCI group.**

| **Metabolites** | **Sub Class** | **m/z** | **Retention time (min)** | **VIP** | **M/C** |
| --- | --- | --- | --- | --- | --- |
| Sodium glycocholate | Bile acids, alcohols and derivatives | 464.302 | 5.89088 | 38.74934 | ↑* |
| Glycocholic acid | Bile acids, alcohols and derivatives | 448.305 | 5.88377 | 11.92091 | ↑* |
| Chenodeoxycholic acid glycine conjugate | Bile acids, alcohols and derivatives | 432.311 | 6.3768 | 9.902031 | ↑* |
| 12-Ketodeoxycholic acid | Bile acids, alcohols and derivatives | 389.269 | 6.60402 | 9.898452 | ↑* |
| N-[(3a,5b,7b)-7-hydroxy-24-oxo-3-(sulfooxy) cholan-24-yl]-Glycine | Bile acids, alcohols and derivatives | 528.264 | 5.94588 | 17.22623 | ↑* |
| 1alpha,25-dihydroxy-2alpha-(3-hydroxypropoxy)-19-norvitamin D3 | Secosteroids | 523.363 | 7.18025 | 9.352958 | ↓* |
| (20S)-24-Hydroxy-19-norgeminivitamin D3 | Secosteroids | 551.394 | 7.52418 | 7.963245 | ↓* |
| 24,24-Difluoro-1,25,26-trihydroxyvitamin D3 | Secosteroids | 455.299 | 6.35653 | 2.083209 | ↓** |
| Atocalcitol | Secosteroids | 539.338 | 5.9665 | 5.420412 | ↓** |
| PI (18:1(9Z)/18:1(9Z)) | Glycerophosphoinositols | 878.575 | 6.36342 | 2.658371 | ↑** |
| PI (20:3(8Z,11Z,14Z)/21:0) | Glycerophosphoinositols | 931.626 | 5.88377 | 18.92676 | ↑* |
| PI (19:1(9Z)/22:2(13Z,16Z)) | Glycerophosphoinositols | 929.61 | 5.89088 | 17.1063 | ↑* |
| Dodecanoic acid | Fatty acids and conjugates | 218.211 | 4.94968 | 1.812207 | ↑* |
| trans-Cinnamic acid | Cinnamic acids | 166.086 | 2.51845 | 2.016708 | ↑** |
| Resolvin D1 | Fatty acids and conjugates | 421.223 | 5.23007 | 1.302614 | ↓** |
| 8-Isoprostaglandin F2a | Eicosanoids | 377.229 | 8.68422 | 1.073063 | ↑** |
| Anandamide (20:l, n-9) | Fatty amides | 376.318 | 8.65672 | 1.634104 | ↑* |
| N-stearoyl valine | Fatty amides | 406.329 | 8.87747 | 8.237223 | ↑* |
| N-docosahexaenoyl GABA | Fatty amides | 414.301 | 6.3768 | 22.94643 | ↑* |
| Astaxanthin diglucoside/ Astaxanthin beta-D-diglucoside | Tetraterpenoids | 919.484 | 5.91838 | 6.109607 | ↓* |
| Lutein | Tetraterpenoids | 613.429 | 6.99662 | 3.072711 | ↓* |
| Tangeraxanthin | Triterpenoids | 529.332 | 6.22587 | 4.083701 | ↓* |
| (-)-Fusicoplagin A | Diterpenoids | 483.295 | 6.39092 | 7.031077 | ↓** |
| 18alpha-Hydroxyglycyrrhetic acid | Triterpenoids | 531.332 | 6.98287 | 1.611783 | ↓* |

M/C: NCI group compared to non-NCI group;

↑: up-regulated;

↓: down-regulated;

**P* < 0.05; ***P* < 0.01.

**Table S7 Altered gut microbiota compositions** **across ARV regimens groups**

| **Altered microbiota** | ***P* value** | **Adjusted *P* value** |
| --- | --- | --- |
| Lechevalieria | 0.165 | 0.687 |
| Faecalibacterium | 0.168 | 0.687 |
| Trichococcus | 0.169 | 0.687 |
| Oribacterium | 0.170 | 0.687 |
| Chujaibacter | 0.170 | 0.687 |
| Thermopolyspora | 0.170 | 0.687 |
| uncultured_compost_bacterium | 0.170 | 0.687 |
| Ambiguous_taxa | 0.170 | 0.687 |
| Lachnospiraceae_UCG-004 | 0.172 | 0.687 |
| Angelakisella | 0.173 | 0.687 |

The model was adjusted with sex, age, CD4 count and sexual preference and BMI; Adjusted *P* value represent comparisons across three ARV regimens (EFV+3TC+TDF; NVP+3TC+AZT; NVP+3TC+TDF); Abbreviations: AZT=lamivudine; EFV=efavirenz; NVP=nevirapine; TDF=tenofovir disoproxil fumarate; 3TC=Lamivudine.

**Table S8. Potential faecal biomarkers and their variation trends across ARV regimens groups.**

| **Metabolites** | **Sub Class** | **m/z** | **Retention time (min)** | **VIP** | **Adjusted P value** |
| --- | --- | --- | --- | --- | --- |
| 29:2(5Z,9Z)(6Br) | Fatty Acids and Conjugates | 511.313 | 7.29035 | 1.45592 | ↓ |
| 3-Hydroxydodecanoic acid | Medium-chain hydroxy acids and derivatives | 234.206 | 5.0371 | 1.49592 | ↑ |
| 2-Hydroxymyristic acid | Fatty acids and conjugates | 262.237 | 5.629283333 | 1.43778 | ↑ |
| 5β-Cholestane-2β,3α,7α,12α,26,27-hexol | Unclassified | 513.342 | 5.7877 | 1.1393 | ↓ |
| Acarbose (M7) | Unclassified | 277.129 | 2.937833333 | 1.53958 | ↓ |
| 2,6,10-Trimethylundecanoic acid | Fatty acids and conjugates | 246.242 | 5.573616667 | 2.13362 | ↑ |
| Lucidenic acid K | Triterpenoids | 517.244 | 5.77395 | 1.34324 | ↓ |
| Atocalcitol | Secosteroids | 539.338 | 5.9665 | 2.54971 | ↓ |
| C19 Sphingosine-1-phosphate | Sphingoid bases | 376.259 | 6.583133333 | 1.34803 | ↑ |
| 12-Ketodeoxycholic acid | Bile acids, alcohols and derivatives | 389.269 | 6.604016667 | 15.8776 | ↑ |

↑: up-regulated; ↓: down-regulated; Adjusted *P* value represent comparisons across three ARV regimens (EFV+3TC+TDF; NVP+3TC+AZT; NVP+3TC+TDF); Abbreviations:AZT=lamivudine;EFV=efavirenz;NVP=nevirapine; TDF=tenofovir disoproxil fumarate; 3TC=Lamivudine. Adjusted *P* value > 0.9.


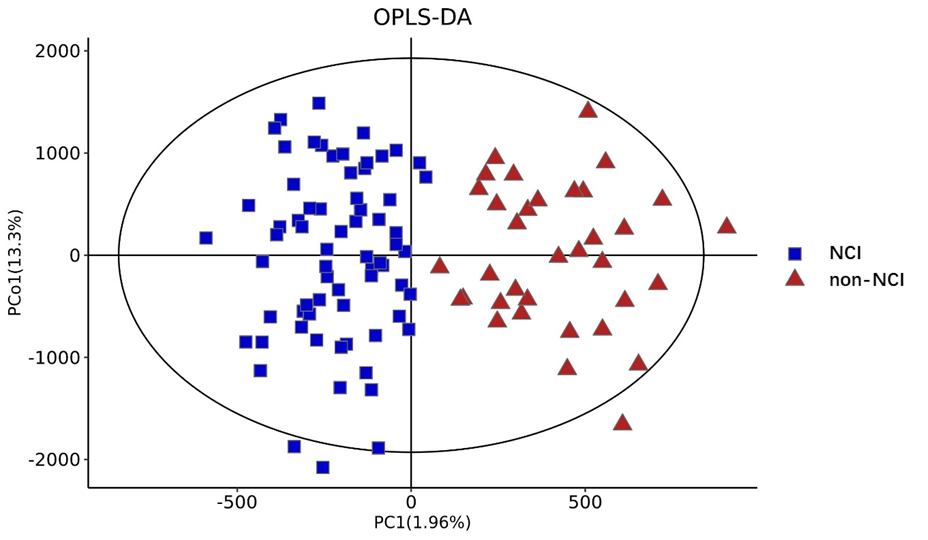


**Figure S3.** OPLS-DA score plots of faecal samples from the NCI and non-NCI groups (R^2^Y=0.782, Q^2^Y=0.215).
